# Catecholamine Variations in Pediatric Gastrointestinal Disorders and Their Neuropsychiatric Expression

**DOI:** 10.3390/biomedicines11102600

**Published:** 2023-09-22

**Authors:** Loredana Matiș, Bogdana Ariana Alexandru, Timea Claudia Ghitea

**Affiliations:** Faculty of Medicine and Pharmacy, Medicine Department, University of Oradea, 410068 Oradea, Romania; matisloredana@yahoo.com (L.M.); ariana.bogdana@gmail.com (B.A.A.)

**Keywords:** catecholamine, neurotransmitter, gastrointestinal disorders, neuropsychological imbalance

## Abstract

The interplay between the central nervous system and the intestinal environment hinges on neural, hormonal, immune, and metabolic reactions. Over decades, significant effort has gone into exploring the link between the digestive system and the brain. The primary objective of this study is to assess catecholamine levels in children with neuropsychiatric disorders. We aim to examine how these levels impact the mental and physical wellbeing of these children, with a specific focus on psychoemotional symptoms and cognitive performance. Our research seeks to identify the significance of modifying neurotransmitter levels in pediatric medical interventions, ultimately striving to reduce mental health risks and enhance children’s future development. A total of 135 individuals were chosen to partake, and they engaged in regular monthly consultations according to established study protocols. Clinical evaluations were conducted in a medical environment, encompassing the observation of constipation, diarrhea, and additional gastrointestinal anomalies not confined to constipation or diarrhea. This entailed the assessment of neurotransmitter imbalances, with a specific focus on dopamine, adrenaline, noradrenaline, and the noradrenaline/adrenaline ratio. Gastrointestinal disorders are indicative of imbalances in catecholamines, with lower gastrointestinal problems being correlated with such imbalances. In subjects with psychiatric disorders, a more pronounced dopamine and noradrenaline/adrenaline ratio was observed, while elevated adrenaline levels were associated with psychoanxiety disorders.

## 1. Introduction

The interaction between the central nervous system and the intestinal environment is founded upon neural, hormonal, immune, and metabolic reactions. For several decades, substantial attention has been dedicated to investigating the correlation between the digestive system and the brain. This unique link between the gastrointestinal tract and the central nervous system has been designated as the “gut–brain axis,” involving a reciprocal exchange [1,2,3]. Recently, considerable emphasis has been placed on comprehending the impact of the gut microbiota on the regulation of the aforementioned gut–brain axis [4]. By activating the hypothalamic–pituitary–adrenal axis and signaling through the vagus nerve, the central nervous system can influence the local enteric nervous system. This cascade results in modifications within the gut environment, encompassing mucus production, gut wall permeability, and the immune response, subsequently affecting the microbiome composition. These shifts can trigger intestinal imbalances, which, in turn, can influence pathological signals to the brain. Hence, a thorough exploration of both afferent and efferent communication pathways, along with their interplay with the microbiome, is essential in order to grasp the pathophysiology of this intricate interaction [5].

Neuropsychiatric disorders stem from diverse origins, leading to initially low success rates in treatment due to challenges in pinpointing precise biological targets [6,7]. Recent studies underscore significant correlations: germ-free mice exhibit lower anxiety under stress; exposure to *E. coli* precedes depression; probiotic treatment alleviates anxiety in laboratory animals [8]. These findings underscore the pivotal role of the microbiome in neuropsychiatric disorders. The burgeoning research into brain–gut–microbiome interconnections holds the potential to unveil the mechanisms underpinning these intricate dynamics [9].

Of paramount importance is dopamine, particularly crucial for coordinating motor functions, memory, learning, concentration, and cognitive performance [10]. Together with serotonin, it exerts mood-modulating effects and governs the reward system, thus shaping motivation and pleasure [11]. A robust interplay between these neurotransmitters is of particular significance. Adrenaline equips the body to tackle heightened demands [12]. It amplifies our respiratory capacity, blood pressure, and heart rate, thereby augmenting the oxygen supply; it enhances our attention and general mental activity, fostering motivation and readiness for action. Moreover, it heightens metabolic activity and temporarily enhances energy availability to muscles and the brain. Conversely, it suppresses digestion and sexual activity [13].

This paper endeavors to assess the repercussions of neurotransmitter level fluctuations on children’s health, following an extended adaptation process. The main aim of this study is to evaluate catecholamine levels in children with neuropsychiatric disorders. Investigating their effects on the mental and physical wellbeing of these children, we emphasized emotional symptoms and cognitive performance. This study investigated the importance of adjusting neurotransmitter levels in pediatric medical interventions. Acknowledging the significance of modulating children’s neurotransmitter levels as an integral facet of pediatric medical intervention can mitigate the risks of mental health deterioration and optimize their future development.

## 2. Materials and Methods

Between 2020 and 2022, a prospective study was conducted at a private nutrition practice in Oradea, Romania, following the guidelines of the World Medical Association’s Declaration of Helsinki.

### 2.1. Patients

The study concentrated on individuals aged 5 to 18, all of whom were diagnosed with metabolic syndrome. Initially, out of 1145 screened individuals, 135 were chosen to participate and they underwent monthly consultations according to the established study protocols. Exclusions from the study included participants over 18 years old, those who declined to participate, and individuals with chronic conditions that could potentially influence the study results.

The required sample size was determined using the appropriate formula for this type of research, which resulted in a specified minimum of 85 cases to achieve a confidence level of 95%. All patients presented with gastrointestinal disorders (constipation, diarrhea) and symptoms other than constipation and diarrhea (such as nausea, flatulence, a feeling of satiety, or gastrointestinal pains), as presented in Table 1, and were prescribed personalized probiotic treatments tailored to address their specific gastrointestinal issues. These recommended probiotics encompassed different combinations and proportions of *bifidobacteria, lactobacilli*, and *saccharomyces* ssp., with formulations excluding gluten or dairy components.

### 2.2. Clinical Analysis

Clinical assessments were performed within a medical setting, observing instances of constipation, diarrhea, and other gastrointestinal issues not categorized as constipation or diarrhea. Thorough patient histories were collected, encompassing details of personal medical backgrounds, medication usage, and tobacco and alcohol consumption, as well as the consumption of other substances with restricted usage.

### 2.3. Paraclinical Analysis

To corroborate the diagnoses, paraclinical evaluations were undertaken. These involved analyzing neurotransmitter disorders, specifically assessing dopamine, adrenaline, noradrenaline, and the noradrenaline-to-adrenaline ratio. These analyses were conducted at an analytical laboratory using enzymatic, colorimetric, and spectrophotometric techniques, alongside immuno-enzymatic tests. Evaluations were performed at the study’s onset and conclusion, using specialized analyses. Specific tests utilizing urine and saliva samples were employed to gauge the presence of stress hormones in the body (CTL and Ortholabor GmbH, 26160 Bad Zwischenahn, Germany).

### 2.4. Statistical Analysis

The study encompassed an analysis of biomarker variations over the research period. Numeric and graphical summaries of individual case profiles were generated, considering changes relative to baseline. The biomarker distributions demonstrated no deviations from normality. Through the employment of a linear mixed model with random effects and an unstructured correlation for repeated measures, alterations over time were modeled. The time of testing was introduced as a fixed effect, initially as a categorical variable for mean change comparisons and, subsequently, as a continuous variable to assess biomarker temporal trends. The relationships between biomarkers were explored via Spearman’s correlations. Statistical analyses were executed using SPSS software (version 20), employing a significance threshold of *p* < 0.05. The fit of the model was evaluated for each biomarker at each time point through an examination of residuals.

## 3. Results

Demographic overview:

Among the total 135 patients, 54 were male (40.0%) and 81 were female (60.0%). Their mean age was 12.57 ± 4.43, ranging from a minimum of 5 years to a maximum of 18 years. The majority of patients hailed from urban areas, constituting 60% of the cohort. From a statistical standpoint, the research groups exhibited parametric characteristics based on skewness and kurtosis testing, with values ranging from −3.00 to +3.00.

**Figure 1:**
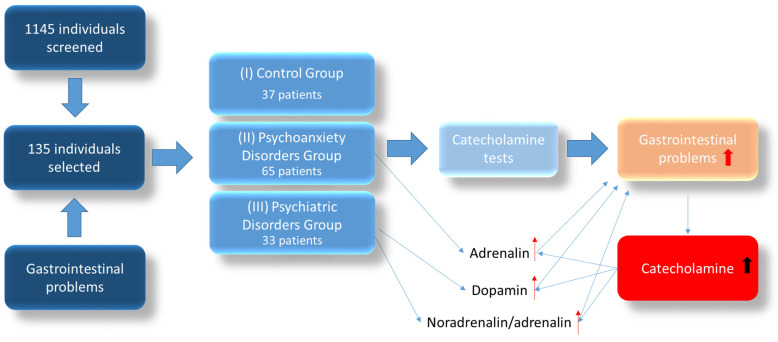
Flow diagram.

The cohort was divided into three distinct groups according to diagnosis by a specialist physician. The applied diet differed in that it was a customized diet combined with a probiotic supplement tailored to address specific gastrointestinal issues. A graphical representation of the patient flow is depicted in Figure 1. This supplement included strains of *Lactobacillus*, *Bifidobacterium*, and *Streptococcus*, totaling 10 billion CFUs, along with supplementation with inulin. In contrast, the control group consisted of individuals without diagnosed disorders who did not receive personalized probiotic therapy. Instead, this group followed a diet that included 18 different cultures, providing a wide-ranging spectrum of probiotics.

In summary, there was:

control group (I) with 37 patients (27.4%);

group with psychoanxiety disorders (II) with 65 patients (48.1%);

group with psychiatric disorders (III) with 33 individuals (24.4%).

### 3.1. Catecholamine Levels

#### 3.1.1. Dopamine

**Figure 2:**
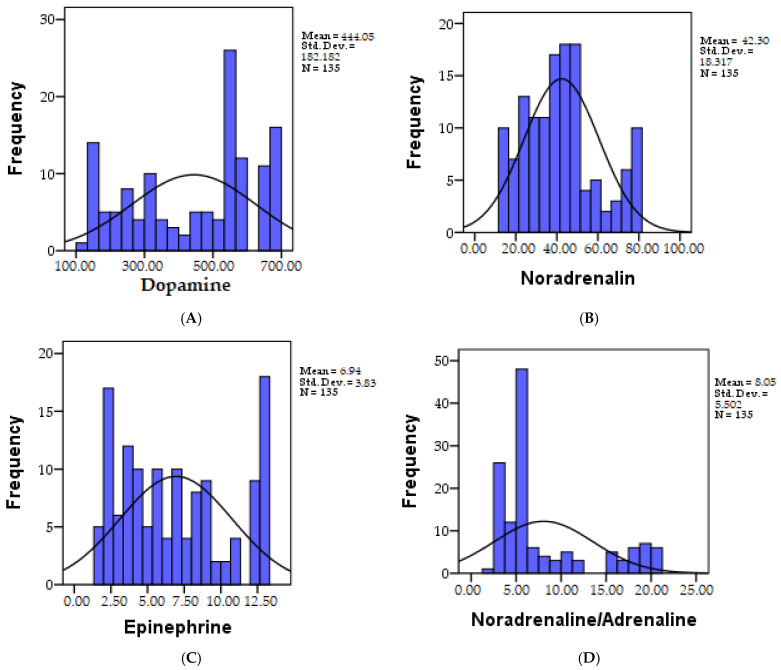
The distribution of the recorded dopamine (**A**), noradrenaline (**B**), adrenaline (**C**), and noradrenaline/adrenaline values (**D**) across the study cohort. Std.Dev = standard deviation, N = number of patients.

Dopamine works as one of the brain’s pivotal neurotransmitters, exerting predominantly stimulating effects. The accepted normal ranges typically span between 125 and 250 µg/g creatinine. Consequently, among the 135 individuals subjected to analysis, it was revealed that 27 individuals (20%) displayed values within the accepted range (125–250 µg/g creatinine) [14], while 108 individuals exhibited values surpassing 250 µg/g creatinine (80.0%). The distribution of recorded dopamine values is visually depicted in Figure 2A.

The initial statistical distinctions among groups, as determined via the ANOVA coefficient (F), indicated significant variations in dopamine levels between the groups (F = 110.587, *p* = 0.001). Subsequently, statistically noteworthy discrepancies (*p* < 0.01) were observed in terms of the differences between batch 1 and batch 2, as well as between batch 1 and batch 3. Conversely, no statistically significant differences were noted between batch 2 and batch 3. Moreover, dopamine levels exhibited no significant differences between patients with psychoanxiety disorders and those with psychiatric disorders.

**Figure 3:**
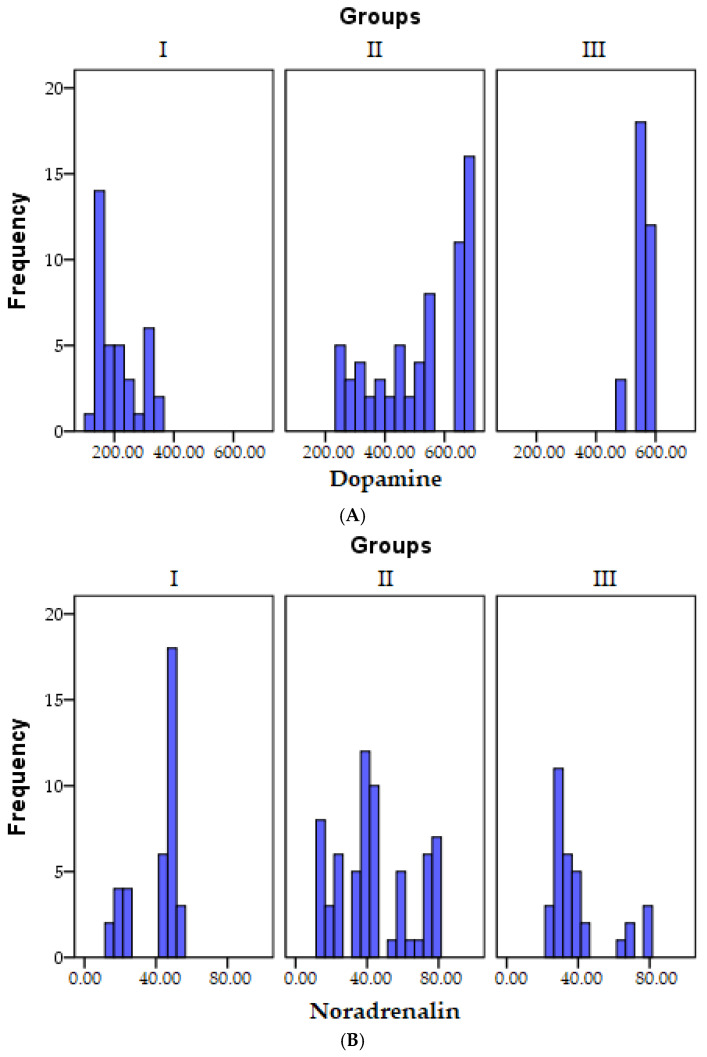

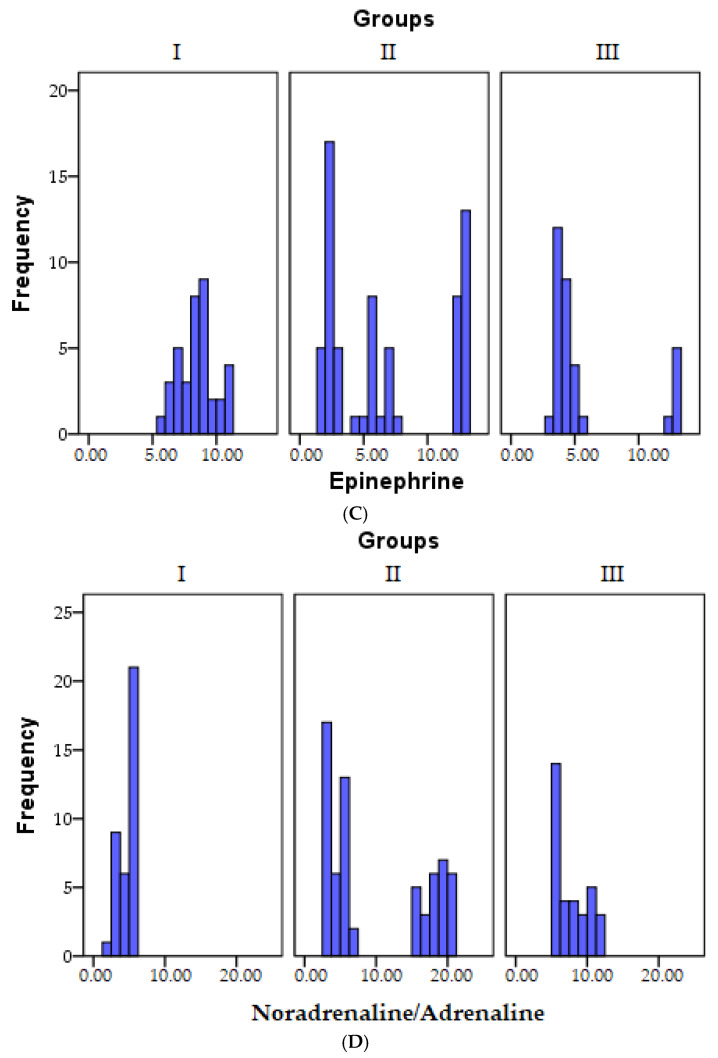
A graphical depiction of the final dopamine (**A**), noradrenaline (**B**), adrenaline (**C**), and noradrenaline/adrenaline results (**D**) corresponding to the three distinct groups (I = control group, II = psychoanxiety disorders group, III = psychiatric disorders group).

Data depicting the distribution of dopamine across the research groups can be observed in Figure 3A.

#### 3.1.2. Noradrenaline

Noradrenaline holds the capacity to elicit effects that encompass heightened blood pressure, enhanced attention, increased alertness, improved concentration, elevated readiness for action, enhanced motivation, and augmented motor functions. It also plays a pivotal role in regulating a diverse spectrum of hormones. The accepted normal range for noradrenaline levels lies within 25–55 µg/g creatinine [15].

In our analysis of 135 individuals, it was ascertained that noradrenaline levels below 25 µg/g creatinine were observed in 27 individuals (20.0%), while normal values, falling within the range of 25–55 µg/g creatinine, were recorded in 81 individuals (60.0%) among the patients. Notably, elevated values exceeding 55 µg/g creatinine were observed in 27 individuals (20.0%). A visual representation of the distribution of recorded noradrenaline values is depicted in Figure 2B.

The initial statistical analysis using the ANOVA coefficient (F) revealed no significant differences between groups in the context of noradrenaline (F = 0.783, *p* = 0.487). Similarly, no statistically significant disparities were observed (*p* > 0.01) when considering the distinctions between group 1 and group 2, as well as group 3. Notably, the levels of noradrenaline did not exhibit significant differentiation between patients with psychoanxiety disorders and those with psychiatric disorders.

A visual representation of the distribution of noradrenaline values within the research groups can be found in Figure 3B.

#### 3.1.3. Epinephrine

Epinephrine (adrenaline) represents the final stage in the catecholamine production pathway. It is primarily synthesized within the adrenal medulla from noradrenaline. The acceptable ranges for adrenaline levels typically span between 3 and 12 µg/g creatinine [16].

The results of our analysis of 135 individuals revealed that 27 individuals (20%) exhibited levels falling below the accepted normal range (<3 µg/g creatinine), while 81 individuals (60.0%) showed levels within the standard range (3–12 µg/g creatinine), and another 27 individuals (20.0%) showcased levels surpassing 12 µg/g creatinine. A visual representation of the distribution of recorded adrenaline values is provided in Figure 2C.

The initial statistical distinctions among groups, as determined by the ANOVA coefficient (F), revealed significant disparities in adrenaline levels between the groups (F = 5.142, *p* = 0.007). Conversely, statistically insignificant differences (*p* > 0.01) were noted in terms of the variations between batch 1 and batch 2, as well as between batch 2 and batch 3. Notably, statistically significant distinctions (*p* < 0.01) were identified between batch 1 and group 3. It is worth noting that adrenaline levels exhibited no significant differentiation between patients with psychoanxiety disorders and those with psychiatric disorders. However, a notable distinction in adrenaline levels was observed between the control group and the group comprising patients with psychiatric disorders.

For a visual representation of the distribution of adrenaline values within the research groups, please refer to Figure 3C.

#### 3.1.4. Noradrenaline/Epinephrine

The noradrenaline/adrenaline ratio serves as a valuable indicator of the overall stress level. A ratio lower than 3 is typically indicative of significant chronic stress. This could arise due to factors such as a deficiency in noradrenaline (resulting from a decreased production of, or deficiencies, in amino acids or micronutrients) or, less commonly, an excess of adrenaline. The accepted standard range for this ratio spans between 3 and 7 µg/g creatinine [17].

Among the 135 individuals examined, the analysis revealed that 11 individuals (8.1%) displayed values below the established normal range (<3 µg/g creatinine), while 79 individuals (58.5%) exhibited values within the normal range (3–7 µg/g creatinine). Additionally, 45 individuals (33.3%) registered values exceeding 7 µg/g creatinine. The distribution of the recorded noradrenaline/adrenaline values can be observed in Figure 2D.

The initial statistical contrasts between groups, assessed through the ANOVA coefficient (F), demonstrated substantial differences in the noradrenaline/adrenaline ratio among the groups (F = 13.667, *p* = 0.001). Additionally, statistically noteworthy distinctions (*p* < 0.01) emerged in relation to the differences between batch 1 and batch 2, as well as between batch 1 and batch 3. Conversely, insignificant differences (*p* > 0.05) were identified between batch 2 and group 3. Notably, the noradrenaline/adrenaline ratio exhibited no significant differentiation between patients with psychoanxiety disorders and those with psychiatric disorders.

Detailed distribution data for the noradrenaline/adrenaline ratio within the research groups can be observed in Figure 3D.

### 3.2. Gastrointestinal Disorders in Patients with Neuropsychiatric Manifestations

Significant disparities were observed initially in the context of both constipation and diarrhea among patients with neuropsychiatric disorders. However, by the culmination of the research period, notable improvements were witnessed, rendering the disparities insignificant. In the case of general gastrointestinal issues, the presence of these problems was evident among all patients at the study’s outset. Remarkably, as the research period concluded, a substantial overall enhancement of 76.3% was documented, resonating across each research group. This improvement was marked by a significant alteration (*p* < 0.05), as illustrated in Table 2.

When we studied the results of the statistical examination encompassing the initial and final assessments of the parameters related to gastrointestinal disorders, notable differences emerged across all three disorders—constipation, diarrhea, and general gastrointestinal issues—yielding *p*-values of less than 0.01.

### 3.3. Correlations

Through Spearman’s correlation analysis, a notable association emerged between the variance in constipation and dopamine levels. As dopamine levels rise, a decrease in constipation becomes evident. Similarly, elevated dopamine levels were linked to a diminished occurrence of diarrhea, which was further corroborated by Spearman’s correlation coefficient (rho) and a *p*-value less than 0.01. Furthermore, an increase in catecholamine levels (noradrenaline, and adrenaline) exhibited a robust positive correlation with the presence of gastrointestinal problems, as presented in Table 3.

**Figure 4:**
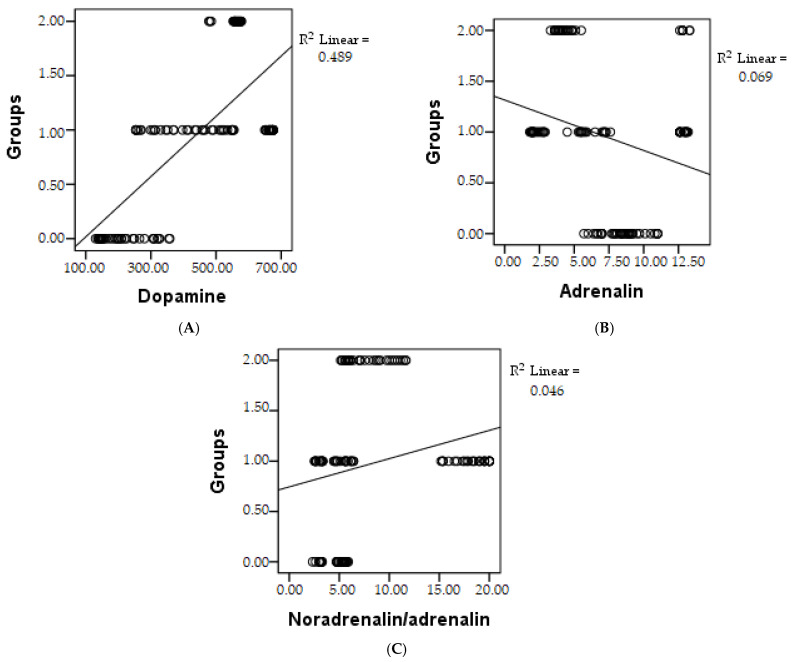
A graphical depiction of the correlation between groups and catecholamines, including dopamine (**A**), adrenaline (**B**), and the noradrenaline/adrenaline ratio (**C**); R^2^ = regression coefficient.

In the examination of the relationships between the existence of neuropsychiatric disorders in patients and the levels of catecholamines, no significant variations in noradrenaline were noted between the groups (*p* > 0.05). However, within the study subgroup consisting of patients with psychiatric disorders (group 3), an ascending trend in dopamine values and the ratio of noradrenaline to adrenaline was evident. Furthermore, a correlation emerged between elevated adrenaline levels and the presence of psychoanxiety disorders, as depicted in Figure 4.

## 4. Discussion

In cases of acute stress, dopamine secretion can increase [18]. Elevated dopamine levels can lead to reduced recuperative abilities and regeneration, resulting in fatigue throughout the day and difficulties in concentration [19]. Additional potential symptoms encompass digestive issues, sleep disturbances, restlessness, and mental disorders such as schizophrenia [20,21,22].

An elevation in dopamine levels can be induced by either acute stress or the onset of chronic stress [18,23]. Moreover, drug consumption can also trigger heightened dopamine levels [14]. Within our study, an elevated dopamine level was found in 80% of patients.

A diminished noradrenaline level can lead to energy depletion, difficulties in concentration, lack of drive, emotional instability, and heightened pain sensitivity, and can potentially contribute to a depletion of other neurotransmitters under conditions of chronic stress [24]. Deficiency in noradrenaline can arise from an insufficient intake of tyrosine or essential nutrients, such as vitamin B6, folic acid, vitamin C, vitamin D [25,26], copper, and magnesium (similar to dopamine deficiency) [27,28].

An excessively high noradrenaline level can lead to symptoms such as anxiety, elevated blood pressure, and increased activity [29]. These outcomes can stem from either acute stress or the onset of chronic stress [30]. Additionally, post-traumatic stress disorder can elevate noradrenaline levels [31]. In our current study, the presence of both the deficiency (observed in 27 individuals) and excess of noradrenaline was identified, although the majority of patients fell within the range of normal values.

A low adrenaline level can result in profound fatigue with a compelling need for sleep, potentially leading to extreme exhaustion. This condition can also manifest as a lack of energy, restlessness, difficulties in concentration, hypotension, and challenges regarding weight loss [32]. Adrenaline deficiency might stem from an inadequate intake of tyrosine or essential nutrients, such as vitamin B6, folic acid, vitamin C, copper, or magnesium, as well as from a lack of vitamin B12 and folic acid (similar to dopamine deficiency) [33].

An augmented adrenaline level can result in challenges falling asleep and feelings of restlessness and anxiety. An excess of adrenaline can be triggered by acute stress or the onset of chronic stress [34,35]. In our study, 40% of patients exhibited imbalances in adrenaline levels, including both elevated and lowered values.

Regarding the noradrenaline/adrenaline ratio, a value between 3 and 7 signifies a balanced proportion of these two messenger substances [36]. It is important to note that this value can also be achieved when both parameters simultaneously increase or decrease. A value ranging from 7 to 12 might result from heightened noradrenaline (as a stress response) or, less commonly, a decrease in adrenaline [37,38]. Ratios exceeding 12 denote a significant imbalance of messenger substances and are often linked with symptoms such as nervousness, sleep difficulties, reduced energy and focus, and even burnout syndrome [39,40,41,42]. In our study, 33.3% of patients exhibited elevated values of the noradrenaline/adrenaline ratio.

Chemical substances called neurotransmitters, such as catecholamines and serotonin, are of fundamental importance in maintaining the internal balance of the human body. Much of the research on these neurotransmitters has focused on their role in the “fight or flight” process, whereby they transmit signals through chemical connections called synapses and regulate blood flow throughout the body [43]. In 2017, Spohn observed that serotonin serves multiple functions within the digestive system, functioning as a regulator of secretion, peristalsis, and absorption at the gastrointestinal level. Furthermore, in the central nervous system, it influences behavioral control and critical neurological functions [44]. The vagus nerve has the capacity to regulate the immune responses, and the gastrointestinal tract serves as a crucial hub for immune system control. Sometimes the immune response can be exaggerated [45]. This nerve plays a vital role in the intricate interplay among the gut, brain, and inflammatory mechanisms. Emerging treatment approaches aim to modulate communication between the brain and gut, known as the brain–gut axis, with therapies such as vagus nerve stimulation [46]. In this study, it was also observed that gastrointestinal disorders indicate an imbalance in catecholamines. Dopamine, a neurotransmitter with a role in nearly all higher cognitive processes, functions through five types of G-protein-coupled receptors. Extensive research has been conducted on dopamine, particularly in terms of its impact on neuronal communication and its association with Parkinson’s disease [47]. However, dopamine receptors can also be found beyond synaptic connections and, furthermore, they are not exclusive to neurons but are also present in various types of cells throughout the mammalian body, both within and outside the central nervous system. These receptors have multiple additional functions, including the regulation of the stress response [48]. In this study, it was noted that individuals with mental disorders tend to experience more frequent increases in dopamine levels. Mehta, in a study published in 2020, observed that an elevated level of noradrenaline is associated with psychosomatic disorders. In our own study, we observed an increase in the levels of noradrenaline and adrenaline in patients with mental disorders. For individuals with anxiety disorders, this response can become dysregulated or excessively sensitive, leading to heightened adrenaline levels even in non-threatening situations. This can result in symptoms such as an elevated heart rate, rapid breathing, sweating, and a sense of impending danger, all of which are characteristic of anxiety [49]. In our study, we observed that elevated adrenaline levels are associated with psychoanxiety disorders.

These aspects of the study encompass complex elements that necessitate comprehensive consideration. One limitation involves the absence of microbiome assessment, and the evaluation of children with varying dietary habits, such as excessive consumption of ultra-processed foods, could yield divergent results. The study’s strength lies in directly assessing catecholamine levels in the context of gastrointestinal problems, thereby offering insights into their intricate interplay in children with neuropsychiatric disorders. The study’s limitation lies in the fact that correlation does not imply causation.

## 5. Conclusions

Gastrointestinal disorders signal a catecholamine imbalance, with a lower incidence of gastrointestinal issues correlated with reduced catecholamine levels.

Group 3, comprising individuals with psychiatric disorders, demonstrates a more frequent increase in dopamine values and the noradrenaline/adrenaline ratio. Additionally, heightened adrenaline levels are linked to psychoanxiety disorders.

## Figures and Tables

**Table 1:** Distribution of patients with gastrointestinal disorders, at the level of the research groups, and in total.

Parameters		Groups						Total	
		I		II		III			
		Count %							
Constipation	No	27	73.0	0	0.0	27	81.8	54	40.0
	Yes	10	27.0	65	100.0	6	18.2	81	60.0
Diarrhea	No	27	73.0	48	73.8	33	100.0	108	80.0
	Yes	10	27.0	17	26.2	0	0.0	27	20.0
Gastrointestinal problems	No	11	29.7	17	26.2	4	12.1	32	23.7
	Yes	26	70.3	48	73.8	29	87.9	103	76.3

I = control group, II = group with psychoanxiety disorders, III = group with psychiatric disorders (III).

**Table 2:** Evaluation of gastrointestinal disorders according to neuropsychiatric manifestations.

Parameters		Groups						*p*	Total	
		I		II		III				
		Count	%	Count	%	Count	%		Count	%
Initial										
Constipation	No	27	73.0	0	0.0	27	81.8	0.001	54	40.0
	Yes	10	27.0	65	100.0	6	18.2		81	60.0
Diarrhea	No	27	73.0	48	73.8	33	100.0	0.004	108	80.0
	Yes	10	27.0	17	26.2	0	0.0		27	20.0
Gastrointestinal problems	No	0	0.0	0	0.0	0	0.0	1.000	0	0.0
	Yes	37	100.0	65	100.0	33	100.0		135	100.0
Final										
Constipation	No	37	100.0	61	93.8	33	100.0	0.110	131	97.0
	Yes	0	0.0	4	6.2	0	0.0		4	3.0
Diarrhea	No	37	100.0	61	93.8	33	100.0	0.110	131	97.0
	Yes	0	0.0	4	6.2	0	0.0		4	3.0
Gastrointestinal problems	No	36	97.3	42	64.6	25	75.8	0.001	103	76.3
	Yes	1	2.7	23	35.4	8	24.2		32	23.7

I = control group, II = group with psychoanxiety disorders, III = group with psychiatric disorders (III), *p* = statistically significant.

**Table 3:** Spearman’s correlation analysis pertaining to the connection between catecholamines and discrepancies in gastrointestinal disorders.

Spearman’s Correlation		Dopamine	Noradrenalin	Adrenalin	Noradrenaline/Adrenaline
Constipation	rho	−0.250 **	−0.083	−0.081	−0.162
	*p*	0.000	0.341	0.352	0.060
Diarrhea	rho	0.307 **	0.632 **	0.070	0.623 **
	*p*	0.000	0.000	0.417	0.000
Gastrointestinal problems	rho	0.057	0.646 **	0.585 **	−0.141
	*p*	0.516	0.000	0.000	0.103
	N	135			

rho = Spearman’s coefficient, *p* = statistically significant, N = number of patients, ** = correlation is significant at the 0.01 level.

## Data Availability

All the data processed in this article are part of the research for a doctoral thesis, which is archived in the aesthetic medical office where the interventions were performed.
